# Pharmacogenetic studies with oral anticoagulants. Genome-wide association studies in vitamin K antagonist and direct oral anticoagulants

**DOI:** 10.18632/oncotarget.25579

**Published:** 2018-06-26

**Authors:** Natalia Cullell, Caty Carrera, Elena Muiño, Nuria Torres, Jerzy Krupinski, Israel Fernandez-Cadenas

**Affiliations:** ^1^ Stroke Pharmacogenomics and Genetics, Fundació Docència i Recerca Mútua Terrassa, Hospital Universitari Mútua de Terrassa, Terrassa, Barcelona, Spain; ^2^ Neurovascular Research Laboratory, Institut de Recerca, Universitat Autònoma de Barcelona, Hospital Vall d’Hebron, Barcelona, Spain; ^3^ Servicio de Neurología, Hospital Universitari Mútua Terrassa, Terrassa, Barcelona, Spain; ^4^ School of Healthcare Science, Manchester Metropolitan University, Manchester, United Kingdom; ^5^ Stroke Pharmacogenomics and Genetics, Institut de Recer ca Hospital de la Santa Creu i Sant Pau, Barcelona, Spain

**Keywords:** VKA, DOACs, pharmacogenetics, GWAs, genetics

## Abstract

Oral anticoagulants (OAs) are the recommended drugs to prevent cardiovascular events and recurrence in patients with atrial fibrillation (AF) and cardioembolic stroke. We conducted a literature search to review the current state of OAs pharmacogenomics, focusing on Genome Wide Association Studies (GWAs) in patients treated with vitamin K antagonists (VKAs) and direct oral anticoagulants (DOACs).

VKAs: Warfarin, acenocoumarol, fluindione and phenprocoumon have long been used, but their interindividual variability and narrow therapeutic/safety ratio makes their dosage difficult. GWAs have been useful in finding genetic variants associated with VKAs response. The main genes involved in VKAs pharmacogenetics are: *VKORC1, CYP2C19* and *CYP4F2.* Variants in these genes have been included in pharmacogenetic algorithms to predict the VKAs dose individually in each patient depending on their genotype and clinical variables.

DOACs: Dabigatran, apixaban, rivaroxaban and edoxaban have been approved for patients with AF. They have stable pharmacokinetics and do not require routine blood checks, thus avoiding most of the drawbacks of VKAs. Except for a GWAs performed in patients treated with dabigatran, there is no Genome Wide pharmacogenomics data for DOACs. Pharmacogenomics could be useful to predict the better clinical response and avoid adverse events in patients treated with anticoagulants, identifying the most appropriate anticoagulant drug for each patient. Current pharmacogenomics data show that the polymorphisms affecting VKAs or DOACs are different, concluding that personalized medicine based on pharmacogenomics could be possible. However, more studies are required to implement personalized medicine in clinical practice with OA and based on pharmacogenetics of DOACs.

## INTRODUCTION

### Oral anticoagulant drugs

Oral anticoagulants (OAs) are recommended drugs to reduce the risk of stroke and systemic embolism in patients with non-valvular atrial fibrillation (NVAF), and to treat and reduce the risk of deep venous thrombosis (DVT) and pulmonary embolism (PE) [1, 2].

OAs can be classified as vitamin K antagonists (VKAs) (warfarin, acenocoumarol, fluindione and phenprocoumon) [3] and direct oral anticoagulants (DOACs): dabigatran, apixaban, rivaroxaban and edoxaban [4, 5].

### Pharmacogenetics of OAs

Genome Wide Association Studies (GWAs) have been very successful in finding genetic risk factors associated with complex diseases or with drug response (pharmacogenomics). This technique uses a general approach which allow a systematic agnostic research of common genetic factors across the whole genome [6, 7].

Furthermore, different GWAs analysis have been published describing variants associated with the interindividual and inter-ethnic variation of VKAs response [6–11] and, recently, also for dabigatran response [12]).

The aim of this review is to describe the genetic factors associated with the response of OAs drugs used in stroke and other cardiovascular diseases prevention and to discuss the future use of OAs pharmacogenomics in the clinical practice.

## MATERIALS AND METHODS

### Literature search

**Figure 1:**
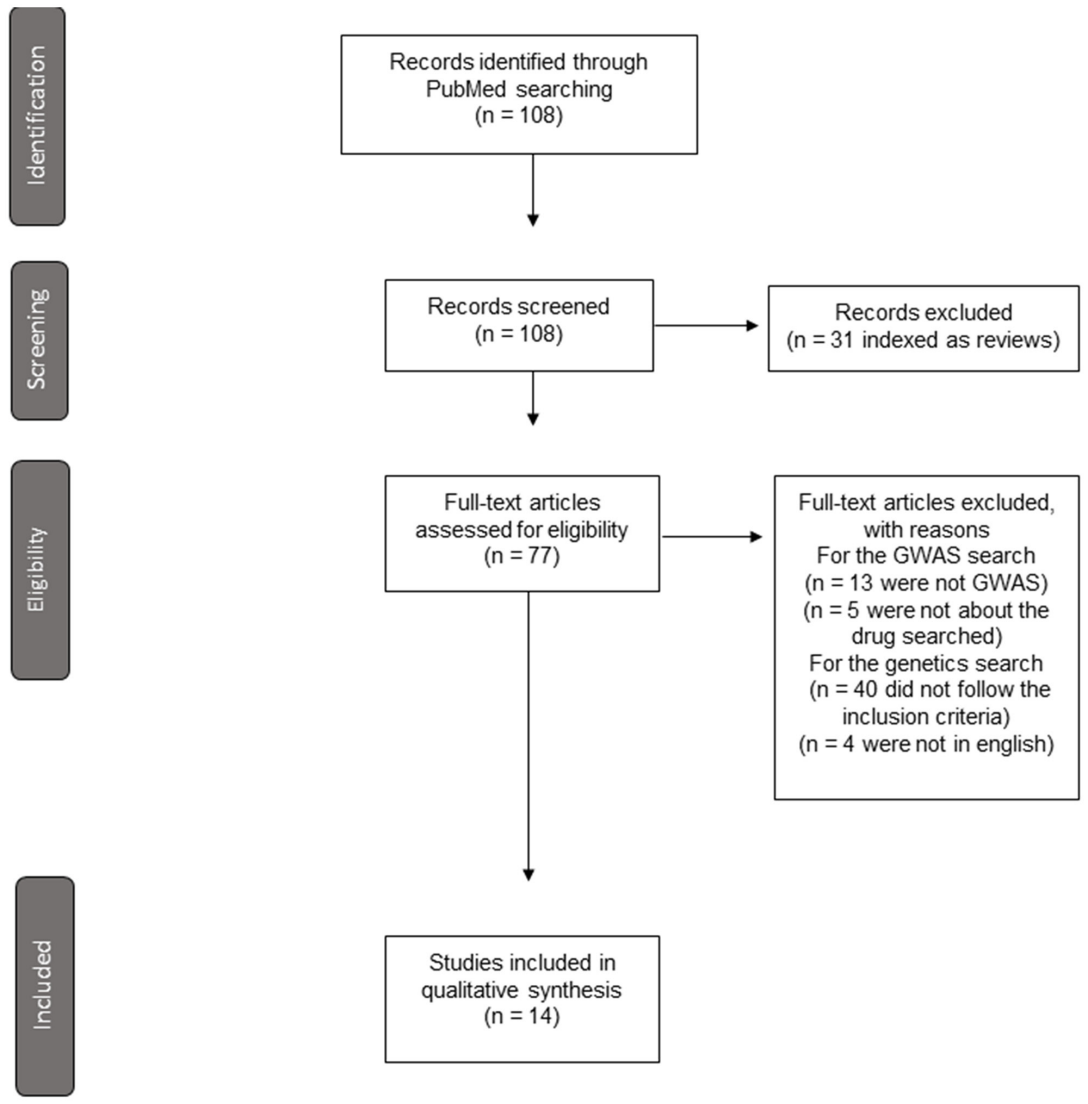
Flow diagram in the selection of articles included in the review

An extensive literature search was performed, up to December 2017, on PubMed with the following key words: ‘GWAs and warfarin’, ‘GWAs and acenocoumarol’, ‘GWAs and fluindione’, ‘GWAs and phenprocoumon’, ‘GWAs and dabigatran’, ‘GWAs and edoxaban’, ‘GWAs and rivaroxaban’ and ‘GWAs and apixaban’. Thirty-eight results were obtained for ‘GWAs and warfarin’, 6 of which were GWAs studies. For ‘GWAs and acenocoumarol’, 4 results were obtained. Only 1 of them was a GWAs study performed on acenocoumarol. One result was obtained for ‘GWAs and phenprocoumon’ which corresponded to a GWAs analysis. Two results were found for ‘GWAs and dabigatran’; only one was a GWAs analysis in patients treated with dabigatran. For the other searches, no results were obtained (Figure 1, Supplementary Table 1).

In the case of drugs for which we did not find GWAS analyses, we searched in PubMed for other genetic studies, based on candidate genes, using the following terms: ‘Genetics and fluindione’, ‘Genetics and edoxaban’, ‘Genetics and rivaroxaban’ and ‘Genetics and apixaban’. We included three candidate gene studies for fluindione, one candidate gene study for edoxaban and one *in vitro* study for apixaban (Figure 1). We included all the original studies written in English including candidate gene analyses in more than 20 patients or *in vitro* studies analyzing the influence of one or more SNPs.

### eQTLs analysis

We searched in GTEx portal (https://www.gtexportal.org/) the first and second most significant eQTLs genes for the SNPs from the published GWAs cited in this review (Supplementary Table 1), in two cases: 1-intragenic SNPs attributed to regulate other genes in the referenced paper; 2-intergenic SNPs (Table 1). We indicated the tissue in which the most significant eQTL for a gene is expressed and also if it is expressed in relevant tissues for the disease.

**Table 1:** Most significant eQTLs for significant intergenic SNPs in AOs GWAs

SNP from GWAS (Supplementary Table 1)	Gene	Near gene (cited in the GWAs)	Most significant eQTLs (GTEx project)	p-value (GTEx project)	Tissue
rs10871454	*STX4*	*VKORC1*	*KAT8*	1.1e-38	Skin^a^
			*VKORC1*	1.5e-30	Liver^b^
rs9923231		*VKORC1*	*KAT8*	1.2e-38	Skin^a^
			*VKORC1*	2.0e-33	Liver^b^
rs12777823		*CYP2C18*	*CYP2C19*	6.9e-31	Esophagus - Mucosa
			*MTND4P19*	3.5e-14	Skin
rs2104162		*CYP2C9*	*CYP2C19*	1.1e-10	Esophagus- Mucosa
			*MTDN4P19*	0.0000069	Esophagus- Mucosa
rs749671	*ZNF646*	*VKORC1*	*KAT8*	1.9e-39	Skin^a^
			*VKORC1*	3.0e-32	Liver^b^
rs4918798		*CYP2C9*	*C10orf129*	1.2e-12	Adipose - Subcutaneous
			*CYP2C8*	0.000077	Breast - Mammary Tissue
rs17126068		*DDHD1*	*RP11-547D23.1*	1.4e-9	Testis
			*DDHD1*	7.0e-7	Muscle - Skeletal
rs889548	*MYST1*	*VKORC1*	*KAT8*	7.1e-43	Skin
			*VKORC1*	1.2e-30	Liver^b^
rs1978487	*MYST1*	*VKORC1*	*KAT8*	1.1e-39	Skin^a^
			*VKORC1*	7.0e-26	Liver^b^
rs749767	*BCKDK*	*VKORC1*	*KAT8*	5.7e-40	Skin^a^
			*VKORC1*	3.9e-27	Liver^b^
rs3862009		*CYP2C19*	*C10orf129*	1.7e-40	Adipose-subcutaneous^a^
			*CYP2C9*	7.2e-9	Adipose-subcutaneous
rs12610189		*CYP4F2*	*AC004791*.2	1.3e-40	Lung^a,b^
			*CYP4F2*	9.3e-11	Skin
rs1998591		*CYP2C18*	*CYP2C19*	1.1e-17	Esophagus - Mucosa
			*C10orf129*	4.1e-10	Nerve - Tibial^a^
rs2104543		*CYP2C18*	*CYP2C19*	1.1e-15	Esophagus - Mucosa
			*C10orf129*	3.8e-10	Nerve - Tibial^a^
rs12772169		*CYP2C18*	*CYP2C19*	2.1e-17	Esophagus - Mucosa
			*C10orf129*	3.1e-10	Esophagus - Mucosa^a^
rs11150604	*ZNF646*	*VKORC1*	*KAT8*	1.1e-38	Skin^a^
			*VKORC1*	1.5e-30	Liver^b^
rs1980889		*CKS2*	*SEMA4D*	1.3e-7	Thyroid
			*CKS2*	0.0000091	Liver
rs746357		*SHC3*	*SEMA4D*	1.3e-7	Thyroid
			*CKS2*	0.0000091	Liver

Enumeration of the most significant eQTLs for SNPs associated with AOs response located in 1) intragenic regions attributed to regulate other genes in the referenced paper; 2-intergenic regions. Column one: SNP ID (from Supplementary Table 1); Column two: gene, for intragenic SNPs; Column three: near gene described in the original GWAs to be the effector gene; Column four: most significant eQTLs from GTEx project. P-value indicated for the most significant eQTL in the gene; Column five: p-values for the most significant eQTL for each gene; Column six: tissues where the most significant eQTLs are expressed.

^a^ Significant eQTL also in arteries (p<10-5); ^b^ Significant eQTL also in whole blood (p<10-5).

eQTL= expression quantitative trait loci; GTEx project= Genotype-Tissue Expression project.

## VITAMIN K ANTAGONIST (VKA)

The first oral anticoagulant drug was discovered in 1941 through its identification as the cause of fatal bleeding in cattle [13]. These animals had eaten spoiled hay made from sweet clover which contained dicoumarol, a type of coumarin [14]. Since then, coumarins have long been used in the pharmaceutical industry to synthetize anticoagulant drugs due to their antagonistic effect on vitamin K [15].

VKAs are used worldwide. Warfarin is extensively used in North America, Scandinavia, the UK and Asian countries. Acenocoumarol and phenprocoumon are used in continental European countries [14, 16, 17]. Fluindione is widely used in France [18].

**Figure 2:**
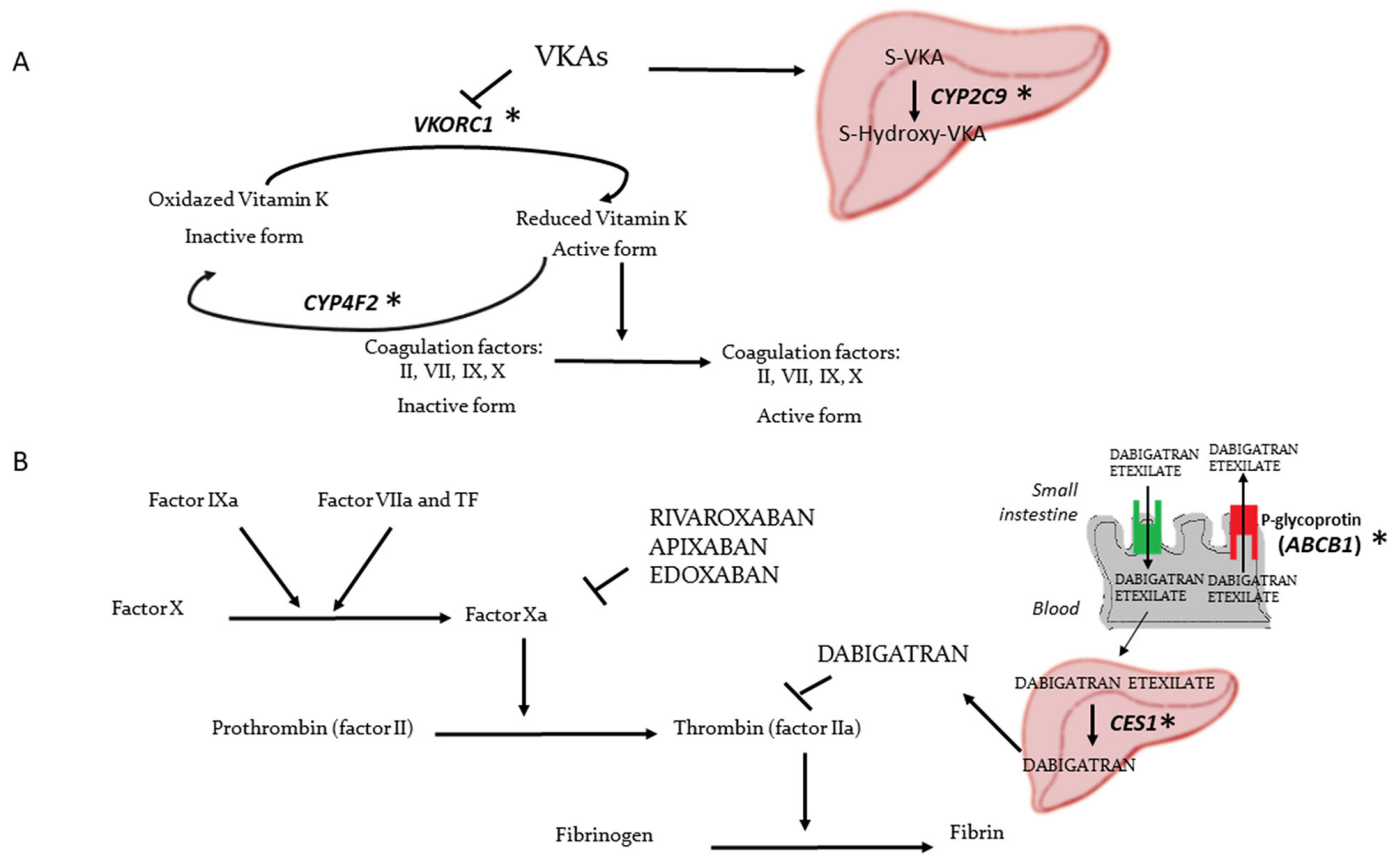
Mechanism of action of the different OAs and polymorphic genes associated with OAs response **(A)** Mechanism of action of VKAs. **(B)** Mechanism of action of DOAC. ^*^Genes with polymorphisms associated with OAs variation in GWAs.

Warfarin and acenocoumarol are 4-hydroxycoumarins, which are vitamin K epoxide reductase (VKOR) inhibitors [14]. They inhibit recycling of the inactive oxidized to the active reduced form of vitamin K, a cofactor involved in activation of coagulation factors II, VII, IX and X [19] (Figure 2A).

VKAs have demonstrated to be effective, preventing 15 deaths and 15 non-fatal strokes per 1,000 patients with non-valvular AF. However, they caused 8 non-fatal major extracranial bleeds [20]. VKAs have a narrow therapeutic/toxic ratio [7, 11, 16, 21, 22], and their daily dose varies between individuals and ethnic groups [23].

Normally, during the first days to weeks of treatment, the correct dose is not achieved as measured by the international normalized ratio (INR). INR values above 3 or 4 increase the bleeding risk, whereas INR values below 2 are associated with low efficacy of VKAs [24]. Patients may therefore be undertreated, increasing the risk of cardioembolism, or overtreated, leading to bleeding, as the therapeutic range is very small [6, 7, 21].

### WARFARIN

Warfarin is a racemic mixture comprised by S and R enantiomers. The S enantiomer has a half-life of 24-33h, whereas R-warfarin has a half-life of 35-58h [14]. S-warfarin is the most potent enantiomer and is almost completely responsible for the anticoagulant effect. This enantiomer is metabolized almost exclusively by the hepatic cytochrome CYP2C9 enzyme [25, 26]. R-warfarin is metabolized by CYP1A1, CYP1A2 and CYP3A4 [27].

The daily dose of warfarin varies between individuals. Doses range from 2 to 10 mg per day [23]. Furthermore, different ethnicities often require different dose of warfarin: Asian populations often have a lower daily dose requirement (3 mg per day) compared to European and African populations (5 mg and 6.5 mg per day, respectively) [23].

Relevant non-genetic factors affecting warfarin dose variance include age, weight, body surface, gender, drug interactions, diseases and the quantity of vitamin K intake [28].

### Pharmacogenetics

The main genetic factors affecting warfarin dose variance found and replicated in different retrospective and prospective studies are SNPs in vitamin K epoxide reductase complex 1 (*VKORC1*) gene, in cytochrome P450 2C9 (*CYP2C9*) gene and in cytochrome P450 4F2 (*CYP4F2*) gene [3, 6, 7, 16, 21, 23, 26, 28–34].

Several GWAs analyses were performed in different populations to analyze the association of genetics with warfarin dose variations [6–10] (Supplementary Table 1). The largest GWAs analysis included 1,508 Japanese patients with the aim of finding associations with warfarin maintenance dose [8]. They found SNPs in *VKORC1* gene to be the most significantly associated with warfarin maintenance dose, followed by polymorphisms in *CYP2C9* and *CYP4F2* genes [8].

### VKORC1

Most studies highlight *VKORC1* as the most important gene in warfarin pharmacogenetics [6–9, 35]. *VKORC1* encodes the catalytic subunit of the vitamin K epoxide reductase complex, which reduces the inactive vitamin K to recycle it to the active form in the endoplasmic reticulum membrane [19, 36]. In the first reported systemic review and meta-analysis on the impact of polymorphisms in or near *VKORC1* gene on warfarin dosage requirement, which included 19 studies and a total of 4,621 patients, the C allele in rs9934438, A allele in rs7294 and G allele in rs9923231 were associated with an increase in the daily warfarin dose requirement [28]. The heterozygous rs7294 CT and rs9923231 GA carriers required a 50% higher dose than the homozygous rs9934438 TT and rs9923231 AA. The homozygous rs9934438 CC and rs9923231 GG carriers required approximately double the dose of rs9934438 TT and rs9923231 AA. The effect of rs7294 SNP on the warfarin dose requirement was less clear. However, rs7294 A carriers required a dose approximately 30% higher than rs7294 GG carriers [28].

In three different GWAs studies, in Swedish, Japanese and African-American populations, the SNP rs9923231 upstream *VKORC1* gene was also found to be significantly associated with the mean warfarin dose (Supplementary Table 1) [7–9]. The Swedish GWAs also found this SNP to be associated with over-anticoagulation (INR > 4) during the first 5 weeks of treatment (Supplementary Table 1) [7]. rs9923231 was linked to rs10871454 (associated with daily maintenance dose in the Caucasian GWAs analyses) [6, 35]. In the GWAs in African-American population [9], rs9934438 in the *VKORC1* gene was also associated with a stable maintenance warfarin dose (Supplementary Table 1), considering the maintenance dose as a stable dose of three or more clinical visits. SNPs in linkage disequilibrium (LD) with rs9923231 on chromosome 16 were also significantly associated with stable warfarin dose [9].

The SNP rs749671, downstream the *VKORC1* gene reached statistical significance in a GWAs study in a Brazilian population (Supplementary Table 1) [10]. The G allele of this SNP was strongly associated with a high warfarin dose requirement [10]. Other SNPs significantly associated with warfarin dose in the *VKORC1* region in this GWAs were rs749670 and rs14235 [10] (Supplementary Table 1).

We have searched the most significant eQTLs of the intragenic and intergenic SNPs attributed to regulate *VKORC1* in the referenced paper. We have found that all these SNPs have eQTLs in *VKORC1* expressed in whole blood, among other tissues (Table 1). These SNPs described to regulate *VKORC1* have also eQTLs in *KAT8*, expressed in arteries (Table 1).

### CYP2C9

The *CYP2C9* gene encodes a member of the superfamily of the cytochrome P450 enzymes important in drug metabolism and lipids synthesis. CYP2C9 enzyme is relevant in the metabolism of almost all the S-warfarin in liver [26, 37]. The most described variants functionally associated with warfarin dosage are CYP2C9^*^2 (rs1799853, R144C) and CYP2C9^*^3 (rs1057910, I359L), which are associated with lower warfarin dose requirements [26]. In a systemic review and meta-analysis including almost 8,000 genotyped individuals, it was reported that the heterozygous genotypes ^*^1^*^2, ^*^1^*^3, ^*^2^*^3 required 19,6%, 33,7% and 56,7%, respectively, lower doses compared to the wild type (WT) genotype ^*^1^*^1. The homozygous genotypes ^*^2^*^2 and ^*^3^*^3 required 36% and 78,1% lower doses compared to ^*^1^*^1 genotype to achieve a stable INR [26]. These genotypes were more influential in individuals without interactions with other drugs [26]. *CYP2C9* variants have an impact on different aspects in warfarin pharmacokinetics. Patients with CYP2C9^*^2 and CYP2C9^*^3 variants require a lower maintenance dose, more time to reach a stabilized dose (mean of 95 days) and have a higher risk of major bleeding compared to patients without these variants. Moreover, these variants are associated with the ratio of above-range INRs [23, 33].

Different GWAs have found other significant variants in *CYP2C9* associated with mean warfarin dose. A GWAs study in a Swedish population confirmed previous results [38] about the significant association between the SNP rs4917639 in *CYP2C9* and the mean warfarin dose given to a patient (Supplementary Table 1). This SNP was in almost complete LD with the composite of CYP2C9^*^2 and CYP2C9^*^3 variants associated with lower warfarin dose requirements [7].

Another GWAs study in Japanese population identified the SNP rs10509680 in the *CYP2C9* gene significantly associated with maintenance dosage of warfarin. The GG genotype of this polymorphism was associated with higher therapeutic dose requirement [8].

Finally, in a GWAs study performed in Brazil, rs4918798 and rs9332238 (the G allele) in or near *CYP2C9* were also associated with high warfarin dose. Rs9332238 and rs4918798, the latter to a lesser degree, were in almost complete LD with CYP2C9^*^2 and CYP2C9^*^3 [10] (Supplementary Table 1).

### CYP4F2

CYP4F2 is another member of the cytochrome P450 enzymes superfamily. This enzyme is a vitamin K1 oxidase important in vitamin K metabolism [19, 30, 39].

In two GWAs, the variant rs2108622 CYP4F2^*^3 (1297G>A) in the *CYP4F2* gene was associated with warfarin dose [7, 8] (Supplementary Table 1). This variant was associated with a higher warfarin dose requirement. This mutation affects the metabolizing function of *CYP4F2* over vitamin K [30]. The cytochrome encoded by the gene *CYP4F2* inhibits vitamin E by hydroxylation of its tocopherol phytyl side chain. This side chain is similar to the side chain of vitamins from the vitamin K group. Thus, it is possible that this cytochrome also inhibits vitamin K, and thereby the activation of vitamin K dependent coagulation factors is reduced when the enzyme works normally [11, 40]. Patients with the CYP4F2^*^3 allele had higher levels of vitamin K1 in the liver and needed an increase of 1–2.5 mg/day in the warfarin dose to achieve proper anticoagulant effect [30].

In relation with ethnic origin, it has been observed that 50% of African-Americans have one of the above described variants in *CYP2C9, VKORC1* or *CYP4F2*, whereas this percentage increases up to 90% in Asians, Caucasians, Hispanics and Ashkenazi Jewish populations [30].

Another GWAs has recently identified two SNPs in *NEDD4* and 2kb downstream *DDHD* (rs2288344 and rs17126068, respectively) associated with warfarin dose. In this GWAs, two SNPs in *ASPH* (rs4379440 and rs17791091) were associated with time in therapeutic range [35] (Supplementary Table 1).

### Implications of warfarin pharmacogenetics

As a consequence of the studies published in relation to the pharmacogenomics of warfarin, in August 2007 the US Food and Drug Administration (FDA) updated the warfarin labelling to incorporate information on the genotyping of *VKORC1* and *CYP2C9* to better adjust the warfarin dose [7, 28]. However, in April 2009, the Centers for Medicare and Medicaid Services (CMS) decided not to routinely pay for the genotyping because they considered there had not been enough evidence supporting the improvement of this genetic test in patient’s health. CMS decided to pay only the genetic test in patients enrolled in specifically designed post-marketing clinical trials [41].

Another consequence of the different pharmacogenetic studies on warfarin was the creation of the International Warfarin Pharmacogenetics Consortium (IWPC) to combine the efforts of warfarin pharmacogenetics researchers. The IWPC included 22 research groups from 11 different countries with total data from more than 5,700 patients treated with warfarin [42].

The described variants in *VKORC1* and *CYP2C9* and the non-genetic factors explain roughly 50% of the interindividual variance in warfarin dose requirement [30]. Thus, around 50% of the warfarin dose variance remains unexplained. Specifically, *VKORC1* polymorphisms explain about 27% (ranging from 15% to 34%) of the variance in the stabilized warfarin dose, whereas the *CYP2C9* polymorphisms contribute on average 12% (ranging from 4 to 20%) [29]. Furthermore, polymorphism in *CYP4F2* (rs2108622) explains roughly 1.5% of warfarin dose variation [7].

Many algorithms have been designed including genetic and non-genetic factors to attempt to predict the appropriate initial or daily stable warfarin dose [16, 21, 42–52].

Gage et al. designed a pharmacogenetic algorithm (PA) using stepwise regression in a multicenter study cohort. They used an additive genetic model, numbering the variant alleles at each locus from 0 to 2. The variables included were weighted according to their ratio of variance on the therapeutic warfarin dose. They tested the effect of several clinical and demographic variables and only retained variables that were significantly independent predictors of warfarin dose in the derivation cohort (N=1,015 patients). The formula was analyzed prospectively in 292 patients from the validation cohort [21]. The pharmacogenetic equation included the 1639G>A *VKORC1* polymorphism, *CYP2C9* ^*^2 and ^*^3 polymorphisms and physiological and clinical factors such as body surface area, age, target INR, amiodarone use, smoker status, African-American race and current thrombosis. This equation explained 53-54% of the warfarin dose variance. When the genetic factors were not included, the equation explained 17-21% of the warfarin dose variability and had higher prediction error [21]. In patients from the validation cohort, the warfarin dose was prescribed prospectively using the PA or an almost equal preliminary version of this algorithm. These patients took a first warfarin dose without accounting for CYP2C9^*^2 and CYP2C9^*^3 alleles. During the 30-day follow-up, 2 patients had a major hemorrhage or PE and 3 patients had symptomatic DVT. They demonstrated the feasibility and safety of the pharmacogenetics algorithm [21].

Recently, two prospective randomized trials were designed to compare patients with initial warfarin dose based on PA or clinical decision [45, 46]. In one of the studies, patients were randomized receiving warfarin by a validated algorithm (96 patients) [53] or *CYP2C9* genotype-adjusted algorithm (95 patients) [46]. They found that the inclusion of *CYP2C9* polymorphisms information decreased minor bleedings and improved the INR control, reaching the first therapeutic INR and stable anticoagulation earlier [46]. In the other study, 200 patients were randomized (101 patients treated using PA and 99 using standard clinical decision). They received warfarin and had at least 1 follow-up INR to prospectively validate the pharmacogenetics-guided dosing formula (determined by a regression equation) [45]. The primary endpoint was the percentage of out-of-range INRs. They did not find significant differences in the ratio of patients with out-of-range INR. However, PA more accurately predicted the individual dose when the patients were divided into subsets: patients without allele variants (WT), patients with a single allele variant and patients with multiple allele variants. The PA was better than the standard decision for predicting the higher average warfarin dose in WT and the lower dose in patients with multiple allele variants. Despite not being statistically significant, total adverse events were lower in patients with dose predicted based on PA than on standard decision [45].

Pharmacogenetic refinement algorithms have been developed to validate whether genotype can refine maintenance dose of warfarin after some days of therapy. One study derived clinical and pharmacogenetics refinement algorithms using INR values on day 4 or 5, clinical factors quantified using stepwise selection and genotype [16]. Variables which achieved statistical significance in the multivariable linear regression model and thereby were maintained were: INR, A allele of the 1639G>A polymorphism in *VKORC1*, CYP2C9^*^2 and CYP2C9^*^3 alleles, prior warfarin dose, age, BSA, stroke, diabetes, race, target INR and use of amiodarone or fluvastatin. The clinical refinement algorithm was similar to the pharmacogenetics algorithm but without the inclusion of genotype and race [16]. In the derivation cohort (N=969), the pharmacogenetics algorithm explained 63% of variation (R^2^), whereas the clinical algorithm had an R^2^ of 48%. The PA was tested in 204 patients of the internal validation cohort who had INR available on day 4th of therapy. The R^2^ was 58%, whereas the R^2^ for clinical algorithm was 43% [16]. Algorithms were also tested in another validation cohort of 105 patients with INR values measured on day 5th. In this case, the R^2^ for the PA was 60%, whereas the R^2^ for the clinical algorithm was 44% [16]. Similar results were obtained when final algorithms were validated in an external validation cohort (N=517 patients with INR measurements on day 4th of therapy; N=438 patients with INR measurements on day 5th of therapy). The authors concluded that PAs were more accurate than clinical algorithms for predicting the maintenance dose of warfarin [16].

PAs developed to predict the initial therapeutic dose of warfarin have shortcomings [21, 45, 46]. They do not indicate how to dose warfarin when the INR response to the therapy is known. Furthermore, in some occasions the time to obtain the genotyping results is too longer. Some experts have argued that when the genotyping of *VKORC1* and *CYP2C9* will be available in practice they may be not relevant or cost-effective. The initiation algorithms have been developed in small populations, with some exception. Thus, the predictive value of these algorithms in large populations could be different [16]. Hence, a large controlled, multicentre and randomized trial seems necessary to quantify the effect of pharmacogenetics algorithms in INR control and in the occurrence of adverse events [21]. In addition, for PA to be used in clinical practice, it is necessary to include the pharmacogenetics technology and knowledge in the clinical infrastructure [34].

The International Warfarin Dose-Refinement (Warfarin DR) Collaboration was created to develop and validate a pharmacogenetics refinement algorithm in an international cohort of patients and determine whether the genotyping predicts the therapeutic dose, also when the INR value is available in the 4^th^ or 5^th^ day after the therapy initiation [16].

### ACENOCOUMAROL

Acenocoumarol is a racemic mixture comprised by S and R enantiomers. S-acenocoumarol is the most potent enantiomer and it is metabolized by the CYP2C9 enzyme, while R-acenocoumarol is metabolized by CYP1A2, CYP3A4, CYP2C9 and CYP2C19. The half-life of S-acenocoumarol is 1.8 hours and the half-life of R-acenocoumarol is 6.6 hours [14]. The slower elimination of the R-enantiomer makes R-acenocoumarol responsible for the anticoagulant effect of this drug. R-acenocoumarol is the clinically most important enantiomer due to the short half-life of S-acenocoumarol [27].

Administration of acenocoumarol is also limited by the narrow therapeutic/toxic profile of this drug and the interindividual and interethnic dose variation. Environmental and genetics factors influence in acenocoumarol dose variation [54].

### Pharmacogenetics

Variations in *VKORC1, CYP2C9* and *CYP4F2* are associated with changes on stabilized acenocoumarol dosage. *VKORC1* and *CYP2C9* genetic variants also correlate with the maintenance dose, the first INR after the initial standard dose, the time until the stable dose is achieved, the time until the correct therapeutic rank is achieved and the ratio of bleeding events of acenocoumarol [11].

Only one GWAs study has been performed on acenocoumarol pharmacogenetics. A large population-based cohort of 1,451 Caucasian patients from the Rotterdam study and 287 individuals from the extended Rotterdam cohort in the replication were analyzed [11].

Near *VKORC1* gene, the SNP rs10871454 in the *Syntaxin-4 (STX4)* gene was significantly associated with acenocoumarol dose variance in this GWAs and subsequently, replicated [11] (Supplementary Table 1). The authors suggested this polymorphism might decrease the hepatic expression of VKORC1 mRNA, decreasing the amount of the drug target [11]. Furthermore, this SNP was in complete LD with the rs9934438 SNP on the *VKORC1* gene that is one of the polymorphisms associated with the dose variation in warfarin treated patients [11]. However, other roles are plausible to explain the association of the SNP in *STX4* and the acenocoumarol dose variance. The protein encoded by *STX4* is a SNARE molecule and SNAREs are involved in endothelial and other cells, such as platelets, secretion. However, its role in platelet exocytosis varies depending on secretary granules types, but appears to be essential for lysosomal release in platelets [55, 56]. Endothelial exocytosis is relevant in thrombosis, hemostasis and inflammation [57, 58].

Regarding *CYP2C9* gene, in this GWAs, the SNP rs4086116 was associated with interindividual variation on stabilized acenocoumarol dosage.

In the same study, the polymorphism rs2108622 in *CYP4F2* was also associated with the acenocoumarol dose when the significant SNPs of *VKORC1* and *CYP2C9* were included in the analysis as covariates [11] (Supplementary Table 1). It has been postulated that the T allele of the rs2108622 variant decreased the enzyme activity, preventing vitamin K inactivation. Consequently, patients with one *CYP4F2* allele variant need an increment of the dose of about 1mg/week per allele [40].

Polymorphisms within and flanking *CYP2C18* and *CYP2C19* gene were also associated with acenocoumarol maintenance dose and replicated in the replication stage [11].

The combination of the CYP2C9^*^3 genotype and the polymorphisms in 1639G>A or 1173C>T in the *VKORC1* gene explained around 50% of the interindividual variability on the anticoagulation effect of acenocoumarol [54]. In addition, the combination of variants in *CYP2C9* and *VKORC1* was strongly associated with severe over-anticoagulation [27]. The addition of the polymorphism rs2108622 (in *CYP4F2*) increased the r^2^ adjusted of the clinical and genetic (*CYP2C9* and *VKORC1*) model for acenocoumarol dosage variation by 1.3%, while the addition of the polymorphism in *CYP2C18* (rs1998591) increased the r^2^ adjusted by 1.2% [11].

### Implications of acenocoumarol pharmacogenetics

Different acenocoumarol pharmacogenetic-guided dosing algorithms have been derived in different cohorts from different populations [17, 59–67]. An observational retrospective study analyzed 8 different acenocoumarol pharmacogenetic algorithms in a cohort of 189 patients [65]. The algorithm which achieved similar doses to the real stable doses was the EU-PACT algorithm [17]. However, considering the patients with over- or under-estimation of the dose and patients correctly classified (deviation from the actual stable dose of ≤20%), the algorithm which classified most patients correctly was the Borobia algorithm [59]. This algorithm correctly classified 40.7% of the 189 patients included [65]. However, an algorithm correctly classifying this percentage of patients remains far from being used in clinical practice.

### PHENPROCOUMON

Phenprocoumon is another coumarin derivate from the VKA family which is also found as a racemic mixture. The S-phenprocoumon is the most potent enantiomer. The half-life of phenprocoumon is 110-130h for the S-enantiomer and 110-125h for the R-enantiomer [14]. Given the long half-life of phenprocoumon, over-anticoagulated patients under this treatment have higher risk of major bleeding [27].

Phenprocoumon is metabolized by CYP3A4 enzyme, which is a non-polymorphic cytochrome [14]. The main role of CYP3A4 in the metabolism of phenprocoumon makes it safer than acenocoumarol and warfarin. 60% of oral phenprocoumon is metabolized, whereas 40% is excreted unchanged. Thus, phenprocoumon could be a better option for those patients that metabolise coumarin poorly [27].

Despite having a narrow therapeutic/toxic profile, patients treated with phenprocoumon achieve more stable INR measurements and require less monitoring [27].

### Pharmacogenetics

It has been suggested than phenprocoumon metabolism is less influenced by the *CYP2C9* genotype, with *VKORC1* having greater relevance. It has been shown that patients with a *CYP2C9* variant, and without *VKORC1* variant alleles, required a 30% lower dose than WT patients. Patients with *CYP2C9* and *VKORC1* allele variants have an increased risk of over-anticoagulation. *CYP2C9* genotype is also associated with delayed phenprocoumon stabilization [27].

A gene candidate study was performed with *VKORC1, CYP2C9* and *CYP4F2* genes. The authors found that an allele variant in *VKORC1* decreased the maintenance dose of phenprocoumon by 4.8 mg/week. An allele variant in *CYP2C9* also decreased the dose by 2.2 mg/week and an allele variant in *CYP4F2* increased the dose by 1.5 mg/week. They generated a clinical-genetic model including age, sex, BMI, target INR, *VKORC1, CYP2C9* and *CYP4F2* genotypes, explaining the 46% of the maintenance dose [68].

There is a GWAs study with 202 patients treated with phenprocoumon. The authors found 32 SNPs in chromosome 16 (within or flanking *VKORC1* gene) significantly associated with phenprocoumon dose. The stronger associations were for the SNPs rs10871454 and rs11150604 (Supplementary Table 1). Both SNPs were in complete LD and were associated with a decrease in the dose. Both SNPs were found replicated in the validation cohort (n=42). Four additional SNPs nominally associated with phenprocoumon dose in chromosome 9 were found. However, they were not replicated [68] (Supplementary Table 1).

### FLUINDIONE

Fluindione is an inandione derivate widely used in France (about the 80% of OA prescription in this country) [69]. Unlike coumarin derivates, fluindione is not found as a racemic mixture [70].

Knowledge of the pharmacokinetics of fluindione is scarce. An intermediate half-life has been described for fluindione, similar to the half-life of the more potent enantiomer of warfarin [18]. One study described a fluindione half-life of 31h [71], while another found a median half-life of 69h [70]. Fluindione is less often displaced by other drugs in their receptors and it has higher affinity to albumin than warfarin [70]. The role of *CYP2C9* is unknown in the fluindione metabolism [72].

The efficacy and, thereby, the dose of fluindione, varies among patients depending on environmental and genetic factors. The individual fluindione dose varies between 5 and 40 mg per day [18, 73, 74]. The interindividual variability of this drug is associated with the same adverse events as with the other VKAs, mainly thromboembolic events and bleedings.

### Pharmacogenetics

No GWAs study was performed in fluindione treated patients. Only some candidate gene analyses have evaluated the genes that could be implicated in the interindividual variability of fluindione. However, it would be necessary to perform GWAs in fluindione to find reliable associations.

In a study including 465 patients with a venous thromboembolic event, several polymorphisms in *VKORC1, CYP2C9, CYP4F2* and *EPHX1* genes were analyzed [72]. The C1173T polymorphism in *VKORC1* gene was associated with different outcomes: the risk of a first INR measure ≥ 2, the mean time to achieve an INR measure in therapeutic range (2-3), the time to have a first INR > 4 (over-anticoagulation). The target dose of fluindione was also associated with the C1173T polymorphism. Patients with a T allele in this polymorphism were more sensitive to fluindione, achieving the first INR in therapeutic range earlier, with an increased risk of over-anticoagulation (achieving a INR value > 4 before) and with a decrease of more than a half the dose of patients with CC genotype [72]. However, these results were not replicated in a validation cohort.

Another study analyzed different variables which could be influencing the fluindione clearance in 24 healthy white patients. The CYP2C9^*^2 and ^*^3 genotype, *VKORC1* 1173 C>T genotype, *CYP1A2* phenotype and body weight were found to be predictors of the fluindione pharmacodynamics and pharmacokinetics [18].

To predict the fluindione dose in an elderly population, one study analyzed 13 polymorphisms in 7 genes (*VKORC1, CYP4F2, EPHX1, CYP2C9, CYP2C19, CYP3A5* and *ABCB1)* potentially involved in the pharmacological effect or in the metabolism/transport of fluindione in 156 patients. The variables body weight, amiodarone intake, *VKORC1, CYP4F2* and *ABCB1* genotypes were included in a prediction model. This model explained 31,5% of the dose variability and the accuracy in the prediction of the dose within 5mg per day of fluindione was 89,7%. The model was validated in 74 patients, obtaining the correct dosing within 5mg per day in 83,3% of the patients. Patients with a variant allele 2kb upstream *VKORC1* (rs9923231) and patients with a variant allele in *ABCB1* 2677 G>T/A required lower doses than WT patients. Interestingly, comparing with coumarin derivates, *CYP2C9* was not associated with fluindione maintenance dose, suggesting the possible use of fluindione in patients hypersensitive to coumarins [74].

There are some differences among the different VKAs. They have different pharmacokinetics, such as different half-life [14]. In pharmacogenetics, there are also some differences in the variants associated with dose variation of the different VKAs. Only in the case of acenocoumarol, polymorphisms flanking *CYP2C18* were identified associated with the acenocoumarol variation dose [27]. Furthermore, CYP2C9^*^2 has different effect in patients treated with acenocoumarol or warfarin. While CYP2C9^*^2 allele decreases the warfarin maintenance dose, this variant does not affect the acenocoumarol dose [27]. In addition, the *CYP2C9* genotype seems to be less relevant for the phenprocoumon and fluindione dose variation than for the other VKAs [27, 74]. Moreover, *CYP1A2* appears to have a more important role for fluindione than for the other VKAs [18].

## DIRECT ORAL ANTICOAGULANTS (DOACS)

Nowadays, 4 DOACs have been developed to try to overcome the drawbacks of VKAs. Rivaroxaban, apixaban and edoxaban are direct oral factor Xa inhibitors, whereas dabigatran is a direct oral thrombin inhibitor [75] (Figure 2B).

DOACs are taken in fixed doses and the coagulation status does not need to be monitored periodically [12]. Furthermore, it has been observed that DOACs have fewer interactions with other drugs such as digoxin, aspirin or nonsteroidal anti-inflammatory drugs or with food [76].

All DOACs have been shown to be safe and effective alternatives to warfarin [77].

Different reviews and meta-analysis have analyzed data from real-world studies, registries and databases in patients with non-valvular AF. In general, they concluded that rivaroxaban is associated with lower rates of ischemic stroke or systemic embolism compared to warfarin [77]. The initiation of rivaroxaban is associated with higher risk of major bleeding compared to apixaban [78] but with lower risk of intracranial bleeding compared to warfarin [79].

Apixaban and dabigatran are associated with lower ratio of major bleeding compared to warfarin [77] and both, apixaban and dabigatran, have similar major bleeding rates [78]. They are also associated with lower ratio of intracranial bleeding compared to warfarin [79, 80]. Both apixaban and dabigatran are associated with lower death rates compared to warfarin [77].

In general, DOACs are similar to warfarin preventing ischemic stroke [77, 80], however with a lower risk of bleeding [77].

### DABIGATRAN

Dabigatran is approved by the FDA and European Medicines Agency (EMA) and indicated for reducing the risk of stroke and systemic embolism in patients with NVAF [1, 2].

Dabigatran is a reversible direct thrombin inhibitor effectively completely converted by the liver esterase CES1 from the oral prodrug dabigatran etexilate to the active drug [12, 81, 82]. Dabigatran etexilate is a substrate of the P-glycoprotein intestinal efflux transporter (encoded by *ABCB1* gene), an efflux pump for xenobiotics. Dabigatran is excreted predominantly by renal pathway (80%) [83]. Maximum plasma concentrations of dabigatran are achieved at 1 to 3 hours after the dose intake [12].

Recently, idarucizumab, a reversal agent for dabigatran action was approved by the FDA and the EMA to treat dabigatran patients with any bleeding or requiring emergency surgery [84].

Several real-world studies with dabigatran have been performed to determine its effectiveness in clinical practice. A meta-analysis of 20 observational real-world studies comparing patients with NVAF treated with dabigatran and with VKA found the incidence of ischemic stroke, major bleeding and mortality was lower for dabigatran than for VKA. However, the risk of gastrointestinal (GI) bleeding was higher for the patients treated with dabigatran (Supplementary Table 2) [85].

Another meta-analysis including 7 observational real-world studies found similar results (Supplementary Table 2). However, they observed a similar rate of stroke between patients treated with dabigatran and patients treated with warfarin. Furthermore, the higher ratio for GI bleeding was potentiated in the elderly subgroup (≥75 years). They included two studies which analyzed the GI risk in patients using dabigatran 110 mg. One of these studies found a similar risk compared with warfarin in patients < 75 years and a higher risk for dabigatran in patients ≥75 years. The other study found lower risk of GI for dabigatran 110 mg compared to warfarin in patients < 75 years. In general, the bleeding outcomes from this meta-analysis were in concordance with the ones in the RE-LY phase III clinical trial [80].

Other datasets included patients with NVAF treated with dabigatran. All of them observed lower risk for major bleeding, intracranial bleeding and death for patients treated with dabigatran compared to patients treated with warfarin (Supplementary Table 2). These associations were stronger for dabigatran 150 mg. Risk of major GI bleeding was similar or higher for patients with dabigatran treatment compared to warfarin. Risk of ischemic stroke was lower or similar between both drugs (Supplementary Table 2) [86–90]. The Danish registry compared the bleeding rates of VKA naïve patients and patients in treatment with VKA switched to dabigatran. They observed that warfarin starters had the highest bleeding rates. Dabigatran 110 mg switchers from VKA had higher rates of bleeding compared to patients continuing warfarin treatment [88–90].

Dabigatran 75 mg was approved in the USA for patients with renal impairment. In an observational study, it was observed that the ratio for stroke, mortality and bleeding was similar in patients treated with dabigatran 75 mg and in patients treated with warfarin. However, the ratio for intracranial bleeding was lower for dabigatran patients. However, most of patients included in this study treated with dabigatran 75 mg did not have renal impairment, so the prescription of this drug was off-label [91, 92].

Dabigatran, like the other DOACs, is taken in a fixed dose. However, there are interindividual differences between patients in blood concentration (estimation of 30% variation for systemic exposure) [12].

The unique GWAs with patients treated with DOACs was developed to identify variants associated with interindividual variability in dabigatran etexilate blood concentration. 1,694 patients of white European ancestry from the RE-LY study treated with dabigatran were analyzed. The polymorphism rs2244613 in the esterase gene *CES1* was associated with trough concentrations (each minor allele was associated with 15% decrease in trough concentrations) and with lower risk of any bleeding. The polymorphisms rs4148738 in the *ABCB1* gene and rs8192935 in *CES1* gene were associated with peak concentrations but not with clinical outcome [12]. However, the results were not analyzed in a replication cohort. Importantly, both genes (*ABCB1* and *CES1*) encode for proteins related to dabigatran pharmacokinetics, which means that variants in genes related to dabigatran absorption and metabolism explain part of the variations in dabigatran concentrations between individuals [12].

Another study in 92 patients with AF has recently evaluated the effect of the three polymorphisms found in the dabigatran GWAs analysis (rs2244613 and rs8192935 in *CES1* gene and rs4148738 in *ABCB1* gene) to analyze the influence of these polymorphisms on the interindividual variation in dabigatran concentration. They found significant association of the SNP rs8192935 in *CES1* gene with dabigatran trough concentration [93].

It is relevant that VKAs and dabigatran have different metabolic pathways. These differences are also observed in pharmacogenetics studies: different polymorphisms in different genes are associated with metabolic variance for each drug. Consequently, future drug decision could be performed in those patients based on their genetic background.

### RIVAROXABAN

Rivaroxaban is a direct oral inhibitor of the Xa factor approved by EMA and FDA, indicated for prevention of stroke and systemic embolism in patients with non-valvular AF [1, 2].

Rivaroxaban does not require coagulation monitoring. The pharmacokinetics of rivaroxaban is dose-proportional and it has high oral bioavailability and maximum plasma concentrations at 3-4 hours after drug intake, which can be affected by the co-administration of food [83]. Rivaroxaban is not recommended in patients with moderate or severe hepatic impairment, as the clearance of rivaroxaban is decreased [83]. Rivaroxaban exhibits little interindividual variability based on age, gender and body weight [94].

This drug is a substrate of the P glycoprotein and is metabolized through CYP3A4/5 and CYP2J2 (two-thirds of rivaroxaban) and CYP independent mechanisms [82, 83, 94].

Approximately 1/3 of unchanged rivaroxaban is eliminated by the kidneys. The other 2/3 of rivaroxaban is metabolized by the liver [95].

There are different studies, registries and databases describing the use of rivaroxaban in real-world for patients with AF [96–103].

In a meta-analysis of 9 studies [96], the results obtained (Supplementary Table 2) were in concordance with the results from the ROCKET-AF clinical trial, confirming the benefit-risk profile of rivaroxaban [97, 99].

The Xantus study, a prospective, observational, phase IV analysis, observed that patients with higher CHADS2 and CHA_2_DS_2_-VASc (a scale for the risk of stroke and systemic embolism) had higher rates of stroke and systemic embolism, major bleeding and death [99, 100].

Different registries and datasets have found results which were similar to the ROCKET-AF phase III clinical trial observations [98, 99, 101] (Supplementary Table 2). In general, the ratio of intracranial bleeding and ischemic stroke were lower for rivaroxaban compared to VKAs [99, 102, 103] (Supplementary Table 2).

There are no GWAs studies performed in patients treated with rivaroxaban. It is possible that polymorphisms in genes involved in the metabolism of the drug could be associated with rivaroxaban blood levels, similar to the results found with warfarin, acenocoumarol and dabigatran. Taking into consideration the proteins associated with the absorption (P-glycoprotein) and metabolism (CYP enzymes) of rivaroxaban, polymorphisms in *ABCB1* gene or *CYP* genes could be associated with rivaroxaban concentration in blood.

### APIXABAN

Apixaban, like rivaroxaban, is a direct oral inhibitor of the Xa factor. Apixaban is indicated by the EMA and FDA for reducing the risk of stroke and systemic embolism in patients with non-valvular AF. It is used in patients with risk factors (previous stroke, high blood pressure, diabetes, heart failure or being ≥75 years old) [1, 2].

The bioavailability of apixaban is around 50% and it reaches the maximum concentration 1-3 hours after intake. Its half-life is between 8 and 15 hours. Roughly 25% of the drug is excreted by the kidneys [104]. Apixaban is also a substrate for the P-glycoprotein transporter and mainly for CYP3A4/5 [83] but also CYP 1A2, 2C8, 2C9, 2C19, 2J2 [94].

A genetic study on the effect of rs4148738 polymorphism in *ABCB1* gene in apixaban concentrations (the same polymorphism of the dabigatran GWAs) showed an association with apixaban peak concentrations [105]. However, it would be necessary to perform GWAs to really assess which genes are important for the interindividual variability.

Currently, there are no GWAs studies in patients treated with apixaban. Taking into account the proteins associated with the metabolism of rivaroxaban, it is possible that polymorphisms in the *ABCB1* gene or *CYP* genes which metabolize apixaban (*CYP3A4/5, CYP 1A2, 2C8, 2C9, 2C19* and *2J2*) could be associated with rivaroxaban blood levels, similar to the results found with warfarin, acenocoumarol or dabigatran.

### EDOXABAN

Edoxaban, such as rivaroxaban and apixaban, is a direct oral inhibitor of factor Xa indicated by the FDA for reducing the risk of stroke and systemic embolism in patients with non-valvular AF [2]. EMA has also approved edoxaban to prevent stroke and systemic embolism in patients with non-valvular AF, but in this case only in patients who have one or more risk factors (previous stroke, high blood pressure, diabetes, heart failure or being ≥ 75 years old) [1].

Edoxaban achieves maximum plasmatic concentrations within 1 to 2 hours after being taken. It has predictable pharmacokinetic profile and 62% oral bioavailability. Edoxaban, as the other DOACs, is also a substrate of the P-glycoprotein. It is metabolized by hydrolysis through a carboxylesterase (CES) and oxidized by the CYP enzyme CYP3A4 [82]. Some 50% of edoxaban is excreted by kidneys [106].

A meta-analysis of 24 real-world studies included patients with AF to analyzed patients treated with a daily dose of edoxaban 30 mg or 60 mg versus placebo (no treatment), aspirin and aspirin plus clopidogrel. They observed how treatment with edoxaban 30 mg decreased the risk for all stroke, ischemic stroke and mortality. Patients treated with edoxaban 30 mg versus patients treated with aspirin plus clopidogrel had a lower risk of intracranial hemorrhage. Edoxaban 60 mg reduced the risk of any stroke and systemic embolism versus placebo, aspirin and aspirin plus clopidogrel. Thus, both edoxaban doses had a positive net clinical benefit compared to antiplatelet treatment or no treatment in the real world [107].

Another study with patients from the Danish nationwide cohort tested the hypothesis that edoxaban had a net clinical benefit (NCB), which is the balance between systemic embolism and intracerebral bleeding, superior to warfarin. Comparing without treatment, warfarin had a NCB of 0.26 prevented events per 100 patients-years, edoxaban 60 mg had a NCB of 0.71 per 100 patients-years and edoxaban 30 mg had a NCB of 0.71 per 100 patients-years. At all CHADS_2_ and CHA_2_DS_2_-VASc score, both edoxaban doses had higher NCB than warfarin. At CHA_2_DS_2_-VASc scores 0 and 1, warfarin had no positive NCB compared to no treatment. In patients with CHADS_2_ and CHA_2_DS_2_-VASc score ≥ 2, edoxaban 60 mg had a better NCB than edoxaban 30 mg and warfarin. At CHA_2_DS_2_-VASc scores 0 and 1, edoxaban 30 mg had superior NCB than edoxaban 60 mg. In patients with HAS-BLED score ≤ 2 (a scale for the risk of major bleeding), both warfarin doses had higher NCB compared to warfarin independently of the CHADS_2_ and CHA_2_DS_2_-VASc scores. In patients with a HAS-BLED score ≥ 3, both edoxaban doses had positive NCB compared to warfarin, in patients with CHADS_2_ and CHA_2_DS_2_-VASc score ≥ 2 [108].

One *in vitro* study was performed with the purpose of determining if two polymorphisms in factor Xa (Ala152Thr and Gly192Arg) were affecting the edoxaban activity. The authors concluded that these mutations do not account for the interindividual variability of edoxaban [109].

There are no GWAs studies performed in patients treated with edoxaban. Studies analyzing the role of *ABCB1* gene, or genes involved in the edoxaban metabolism (*CYP3A4* or *CES* such as dabigatran), and the response to edoxaban could be interesting.

## CONCLUSIONS

Variants in *CYP2C9*, *VKORC1* and *CYP4F2* are associated with VKAs dose variation between individuals. However, these variants together with non-genetic factors explain about 50% of the interindividual dose variation [7, 30]. Further analyses with more patients are necessary to find other genetic variations associated with dose variations or with vascular events and dosing treatment. However, other mechanisms could explain the remaining percentage of variation, such as rare mutations, epigenetics mechanisms and environmental factors. To find rare mutations, it would be necessary to perform exome sequencing or whole genome sequencing as was done for statin drugs [7]. For epigenetics, some mechanisms such as DNA methylation could be implicated in OAs dose variation. Some studies have found how DNA methylation modifications could be influencing the effects of antiplatelet drugs [110, 111].

Diverse pharmacogenetics algorithms have been developed, including polymorphisms in *CYP2C9* and *VKORC1* together with clinical variables to predict the initial dose of warfarin. The different PAs improved the accuracy and efficiency of warfarin dose initiation. However, the results are controversial in the reduction of out-of-range INRs. Some studies observed that genotyping variants in *CYP2C9* improves INR control [46], whereas other studies did not find the usefulness of these algorithms to control INR [21, 45]. Although the percentage of out-of-range INRs is not statistically significant among patients treated according to PA or clinical/standard decision in these studies, PA more accurately predicted the individual dose depending on the subset of patients (WT, with one single allele variant, with multiple allele variants). The percentage of each of these subsets of patients could differ between studies and cause the mentioned differences among studies. Moreover, *VKORC1* have not been genotyped in all studies and could modify the results and conclusions. Probably larger number of patients are necessary to achieve higher statistically power to observe differences among PA and clinical algorithms. Moreover, the genetic variants identified to date explain only a proportion of the drug response, thus more genetic variants can contribute to increasing the percentage of drug variability and may be included in pharmacogenetics algorithms [34].

To improve the main shortcomings of VKA, mainly the frequent monitoring, DOACs have been approved for the prevention of stroke recurrence and systemic embolism in patients having suffered a previous stroke with non-valvular AF, to treat DVT or PE. DOACs have a better efficacy-safety profile compared to warfarin (Daiichi Sankyo Europe GmbH 2014). Patients treated with DOACs have a lower ratio of bleeding events (rivaroxaban, apixaban and edoxaban) and life-threatening bleeding (dabigatran). Furthermore, the use of DOACs reduces intracranial bleeding compared to warfarin. In patients with AF, the results from the different pivotal clinical trials suggest that DOACs improve the outcomes of these patients [15]. Altogether, the information on the four DOACs from the clinical trials shows that they are not inferior to warfarin in terms of efficacy, although they seem to reduce bleeding events, improving their safety. However, DOACs have greater acquisition costs. Thus, greater cost versus greater efficacy/safety profile and avoidance of monitoring have to be balanced [112]. A review of the use of DOACs in an elderly population with non-valvular AF demonstrated that they are beneficial and lead to a reduction of stroke risk in this population [76].

Due to the lack of GWAs with DOACs (only one) [12], it would be important to perform pharmacogenetic studies based on a GWAs approach. It is possible that some genes could be associated with the metabolism of all or almost all DOACs, such as *ABCB1,* which is involved in the transport of all DOACs, or *CYP3A4,* involved in the metabolism of all DOACs with the exception of dabigatran [82, 94]. Furthermore, it could be interesting to assess whether there are polymorphisms associated with the response to the different DOACs in terms of efficacy (risk of stroke or systemic embolism) and safety (risk of bleeding).

Pharmacogenetics could be useful to identify the more appropriate anticoagulant for each specific patient, avoiding adverse events such as any bleeding or stroke recurrence. VKAs and DOACs do not share the same mechanism of action. This fact distinguishes both kinds of anticoagulant, meaning that VKAs could be more suitable for one individual but DOACs for another. Moreover, VKAs and dabigatran have different metabolic pathways and as different polymorphisms affect metabolism of both kind of drugs, knowing the specific genotype of each individual before starting anticoagulant treatment could help avoid adverse events. For instance, a sub-study of the ENGAGE-AF-TIMI 48 trial demonstrated that patients with the *CYP2C9* and *VKORC1* variants can benefit more from edoxaban compared to warfarin [113, 114]. Therefore, a personalized medicine for oral anticoagulants use is possible based on the fact that DOACs and VKAs involve different metabolic pathways and GWA studies confirm that different genetic risk factors are associated with the response of both types of drug.

## FUTURE

It is expected that around year 2020 the results of large randomized clinical trials on the incorporation of pharmacogenetics algorithms to guide VKAs dosage will become available. Furthermore, by this time, it is likely that genotyping will be quickly and more cost-effective. It is expected that the use of pharmacogenetics algorithms for vitamin K inhibitors will reduce adverse reactions to these drugs [3].

Pharmacogenetic studies with DOACs would help to choose the most appropriate anticoagulant treatment for each patient. Thus, pharmacogenetics studies have to be translational, to include genotyping in clinical practice and achieve a more personalized medicine, as happens with other kind of treatments, such as in cancer [115, 116].

The applicability of pharmacogenetics in clinical practice has been mainly demonstrated with cancer treatments. Nowadays, there are cancer drugs which are dosed following pharmacogenetic tests. The genotyping of thiopurine S-methyltransferase (*TPMT)* in acute lymphoblastic leukemia (ALL) patients before treatment with 6-mercaptopurine is useful to select the correct dose based on genetics before the first drug intake. After changing the label for 6-mercaptopurine in 2004, some hospitals routinely started to order *TPMT* genotyping before initiation of the treatment [117].

In advanced colorectal cancer, irinotecan is the most widely-used drug. However, polymorphisms in diphosphate-glucuronosyltransferase 1A1 (*UGT1A1)* have been found to be associated with the risk of developing hematological and/or digestive toxicities. Thus, dose reduction was recommended in patients with the genotype ^*^28/^*^28. In United States, the FDA recommended genotyped the *UGT1A1* ^*^28 variant before irinotecan prescription. In Europe, a Dutch workgroup and the French National Thesaurus of Digestive Oncology recommended dose reduction in ^*^28/^*^28 patients [118]. A genotyping test has been generated to facilitate the detection of this genetic variation *UGT1A1* (Mayo Medical Laboratory) [117, 119].

Other genes associated with adverse drug events with potential benefits for use in clinical practice could be: *CYP2D6* and tamoxifen for breast cancer, *DPYD* and fluoropyrimidine antimetabolite 5-fluorouracil (5-FU) for colon cancer, *SLCO1B1* and Simvastatin for the reduction of cholesterol levels, HLA-A^*^33:03 and ticlopidine for secondary prevention of atherothrombosis [117, 120].

Current medicine could incorporate pharmacogenetics to offer patients an effective personalized medicine by adjusting treatment and dosage in those cases with a proven genetic association based on GWAs analysis and subsequent approval by the FDA or the European authorities. This way, adverse events related with lack or excess of drug effect could be decreased.

## LIMITATIONS

This review has some limitations. First, the idea was to revise and analyze all the GWAS published for the different OAs. However, for some of them (fluindione, edoxaban, rivaroxaban and apixaban), there are no studies that have performed GWAS analysis with these drugs. In these cases, other genetic studies based on gene candidates were reviewed. Nevertheless, these kind of studies are biased, as they are based on a previous hypothesis and do not replicate the results in independent populations. Thus, their reliability is limited. It would be necessary to perform a GWAs approach to really identify genes involved in the secondary response to these drugs.

Second, for many polymorphisms found associated with the effect of different OAs is assumed that the effector gene is the closest to the mutations. However, this is not always true and *in vitro* or *in silico* functional analysis have to be performed to demonstrate the effector gene for each specific mutation.

## SUPPLEMENTARY MATERIALS TABLES







## Abbreviations

OAs: Oral anticoagulants
AF: Atrial fibrillation
GWAs: Genome Wide Association Studies
VKAs: vitamin K antagonists
DOACs: direct oral anticoagulants
NVAF: nonvalvular atrial fibrillation
DVT: deep venous thrombosis
PE: pulmonary embolism
*VKORC1*: *vitamin K epoxide reductase complex 1*
*CYP2C9*: *cytochrome P450 2C9*
*CYP4F2*: *cytochrome P450 4F2*
FDA: US Food and Drug Administration
CMS: Centers for Medicare and Medicaid Services
IWPC: International Warfarin Pharmacogenetics Consortium
PA: pharmacogenetic algorithm
WT: wild type
Warfarin DR: international Warfarin Dose-Refinement
*STX4*: *Syntaxin-4*
EMA: European Medicines Agency
GI: gastrointestinal
CES: carboxylesterase
NCB: net clinical benefit
BMI: body mass index
TPMT: thiopurine S-methyltransferase
ALL: acute lymphoblastic leukemia
TCL1A: T-cell leukemia 1A
