# Supplementary material for: Pharmacogenetic studies with oral anticoagulants. Genome-wide association studies in vitamin K antagonist and direct oral anticoagulants
Source: Oncotarget. 2018 Jun 26;9(49):29238–58. doi: 10.18632/oncotarget.25579 (PMC6044386; doi:10.18632/oncotarget.25579)
Supplement: Supplementary file 1 [file oncotarget-09-29238-s001.pdf]

# Pharmacogenetic studies with oral anticoagulants. Genome-wide association studies in vitamin K antagonist and direct oral anticoagulants

## SUPPLEMENTARY MATERIALS

**Supplementary Table 1: GWAs performed with the different oral anticoagulants, showing the variants significantly associated in the discovery analysis and in the replication.**

**See Supplementary File 1**

**Supplementary Table 2: Real-world studies with DOACs.**

**See Supplementary File 2**
