# Supplementary material for: Pharmacogenetic studies with oral anticoagulants. Genome-wide association studies in vitamin K antagonist and direct oral anticoagulants
Source: Oncotarget. 2018 Jun 26;9(49):29238–58. doi: 10.18632/oncotarget.25579 (PMC6044386; doi:10.18632/oncotarget.25579)
Supplement: Supplementary file 2 [file oncotarget-09-29238-s002.docx]

| **Outcome analyzed** | Daily maintenance warfarin  Dose^c^. | | | | | | | Mean warfarin dose (mg/week) | | | | | |
| --- | --- | --- | --- | --- | --- | --- | --- | --- | --- | --- | --- | --- | --- |
| **P-value COMBI** | 4.7x10^-34^ |  |  | 6.2x 10^-12^ | 1.8x10^-5^ | 8.6x10^-7^ |  | 2.7x10^-181 h^ | 2.6x10^-79 h^ | 1.1x10^-31 h^ | 3.3x10^-10 h^ |  | Over-anticoagulation (INR>4) during first 5 weeks of treatment. |
| **P-value** | 1.0x10^-22^ |  |  | 1.2x10^-8^ | 6.7x10^-2^ | 0.002 |  | 5x10^-62 h^ | 2.3x10^-26 h^ | 5.5x10^-7 h^ | ^g^ 0.0029 ^h^ |  |  |
| **Replication cohort** | 2 cohorts: n=297; n=92 ^a^ | | | | | | | n=588 ^a^ | | | | | |
| **Other significant SNPs in the region** | 5 |  |  |  |  |  |  |  |  |  |  |  |  |
| **P-value MULTI** |  |  |  |  |  | 0.003^b^ | 1.5x10^-6 b^ | 7.3x10^-97 d^ | 1.2x10^-24 d^  3.8x10^-43^ ^e^ | 2.4x10^-14 d^  1.0x10^-15 e^  1.4x10^-26 f^ | 4.8x10^-6 d^  4.6x10^-7 e^  8.3x10-8 f  8.3x10^-10^ g |  |  |
| **P-value**  **UNI** | 6.2x10^-13^ | 9.7x10^-5^ | 2.0x10^-4^ | 8.3x10^-5^ | 6.6x10^-7^ | 9.2x10^-5^ |  | 5.4x10^-78^ | 4.5x10^-17^ | 8.8x10^-13^ | 1.6x10^-5^ | <3.1x10^-31^ | 8.9x10^-9 i^ |
| **Gene** | *STX4 (downstream* 60 kb *VKORC1)* | *CYP2C9* | *CYP2C9* | *CYP2C9* | *FGFBP2* | *CACNA1C* | *LOC105371042* | *VKORC1 (upstream 2kb)* | *CYP2C9* | *CYP2C9* | *CYP4F2* | *CYP2C9* | *VKORC1* *(upstream 2kb)* |
| **HGVS Names** | *NC_000016.10:g.31036758C>T* | *NC_000010.10:g.96725535A>C* | *NC_000010.10:g.96741053A>C* | *NC_000010.10:g.96707202C>T* | *NC_000004.11:g.15963673G>A* | *NC_000012.11:g.2729632A>G* | NC_000016.10:g.1134967G>A | *NC_000016.10:g.31096368C>A* | *NC_000010.10:g.96741053A>C* | *NC_000010.10:g.96702047C>T* | *NC_000019.10:g.15879621C>T* | *NC_000010.10:g.96725535A>C* | *NC_000016.10:g.31096368C>A* |
| **SNPs** | rs10871454  (r^2^=1 with rs9923231) | rs4917639  (*2/*3 tag) | rs1057910-*CYP2C9*3* | rs4086116  (r2=1 with rs4917639) | rs2286461 | rs216013 | rs11865472 | rs9923231 | rs1057910 (*CYP2C9*3*) | rs1799853 (*CYP2C9*2*) | rs2108622 | rs4917639 | rs9923231 |
| **Discovery cohort** | 181 white patients ^a^ | | | | | | | 1,053 Swedish subjects^a^ | | | | | |
| **Treatment** | WAR | | | | | | | WAR | | | | | |
| **Ref.** | [6] | | | | | | | [7] | | | | | |

| **Outcome analyzed** | Daily maintenance  dose (low dose and high dose) | | Stable maintenance WAR dose ^l^ | | | | Low stable WAR dose or high stable WAR dose | | | | | | |
| --- | --- | --- | --- | --- | --- | --- | --- | --- | --- | --- | --- | --- | --- |
| **P-value COMBI** |  |  | 2.57x10^-8^ |  |  | 4.5×10^-^¹² ^k^ |  |  |  |  |  |  |  |
| **P-value** |  |  | 4.97x10^-4^ |  |  | 5.04x10^-5 k^ |  |  |  |  |  |  |  |
| **Replication cohort** | No | No | Yes (n=444). | Yes (n=432). | | | n=798 European ancestry; n=1611 Japanese ancestry and n=191 African-American ancestry^a^ | | | | | | |
| **Other significant SNPs in the region** | 27 | 19 with p-values < 1x10^-5^ |  | 14 |  |  | Multiple significant SNPs |  | Multiple significant SNPs | | | | |
| **P-value MULTI** |  |  | 8.67x10^-6 j^ |  |  | 1.51x10^-8 k^ | 9.5x10^-8 k^ | 1.08x10^-33 m^ | 3.38x10^-33 m^ | 4.4x10^-13 m^ | 2.3x10^-13 m^ | 2.1x10^-7 m^ | 1.8x10^-5 m^ |
| **P-value**  **UNI** | 8.65x10^-31^ | 3.84x10^-7^ |  | 2.08x10^-9^ | 2.08x10^-9^ | 9.67x10^-7^ | 8.02x10^-7^ |  |  |  |  |  |  |
| **Gene** | *VKORC1*  *(upstream 2kb)* | *CYP2C9* | *CYP4F2* | *VKORC1*  *(upstream 2kb)* | *VKORC1* | *CYP2C18*  *(upstream 40kb)* | *CYP2C9 (upstream 30kb)* | *ZNF646 (downstream 10kb VKORC1)* | *VKORC1 (upstream 2kb)* | *CYP2C9* | *CYP2C9 (downstream 1kb)* | *CYP2C9* | *CYP2C9* |
| **HGVS Names** | *NC_000016.10:g.31096368C>A* | *NC_000010.10:g.96734339G>T* | *NC_000019.10:g.15879621C>T* | *NC_000016.10:g.31096368C>A* | *NC_000016.10:g.31093557G>A* | *NC_000010.10:g.96405502G>A* | *NC_000010.10:g.96405502G>A* | *NC_000016.10:g.31077026G>A* | *NC_000016.10:g.31096368C>A* | *NC_000010.10:g.96748492G>A* | *NC_000010.10:g.96750543C>A* | *NC_000010.10:g.96702047C>T* | *NC_000010.10:g.96741053A>C* |
| **SNPs** | rs9923231  (r2=0.992 with rs10871454) | rs10509680 | rs2108622 | rs9923231 | rs9934438 | rs12777823 | rs2104162 | rs749671 | rs9923231 | rs9332238 (LD with *CYP2C9*2* and **3*). | rs4918798 | *CYP2C9*2* | *CYP2C9*3* |
| **Discovery cohort** | 1,508 Japanese patients (807 low WAR dose /701 high therapeutic dose) ^a^ | | 533 (about 85% African ancestry)  ^a^ | | | | 180 low WAR dose and n=187 high WAR dose from Brazil ^a^ | | | | | | |
| **Treatment** | WAR | | WAR | | | | WAR | | | | | | |
| **Ref** | [8] | | [9] | | | | [10] | | | | | | |

| **Outcome analyzed** | WAR maintenance dose | | | | | | | | Time in therapeutic range | |
| --- | --- | --- | --- | --- | --- | --- | --- | --- | --- | --- |
| **P-value COMBI** |  |  |  |  |  |  |  |  |  |  |
| **P-value** |  |  |  |  |  |  |  |  |  |  |
| **Replication cohort** |  |  |  |  |  |  |  |  |  |  |
| **Other significant SNPs in the region** | 36 | | 27 | | | |  |  |  |  |
| **P-value MULTI** | 4.4x10^-64 o^ | 7.26x10^-64 o^ | 5.64x10^-32 o^ | 8.47x10^-32 o^ | 1.77x10^-22^ ^o^ | 4.98x10^-11 o^ | 4.14x10^-8^ ^p^ | 3.96x10^-8 p^ | 5.00x10^-8 q^ | 4.13x10^-9 q^ |
| **P-value**  **UNI** |  |  |  |  |  |  |  |  |  |  |
| **Gene** | *STX4 (downstream* 60 kb *VKORC1)* | *VKORC1*  *(upstream 2kb)* | *CYP2C9* | *CYP2C9* | *CYP2C9* | *CYP2C9* | *DDHD1* (downstream 2kb) | *NEDD4* | *ASPH* | *ASPH* |
| **HGVS Names** | *NC_000016.10:g.31036758C>T* | *NC_000016.10:g.31096368C>A* | *NC_000010.10:g.96743943G>A* | *NC_000010.10:g.96725535A>C* | *NC_000010.10:g.96741053A>C* | *NC_000010.10:g.96702047C>T* | *NC_000014.8:g.53499897A>G* | *NC_000015.10:g.55830149T>G* | *NC_000008.10:g.62465814G>T* | *NC_000008.10:g.62463597G>T* |
| **SNPs** | rs10871454  (r2=0.994 with rs9923231) | rs9923231 | rs9332220  (r^2^=1, with rs4917639, which tags *2 and *3) | rs4917639 | rs1057910(*3) | rs1799853(*2) | rs17126068 | rs2288344 | rs4379440 | rs17791091 |
| **Discovery cohort** | 982 patients ^n^ | | | | | | | | | |
| **Treatment** | WAR | | | | | | | | | |
| **Ref** | [34] | | | | | | | | | |

| **Outcome analyzed** |  | | | | | | | | | | | | | | | | | | | | | | | First INR after initial dosage of ACE |
| --- | --- | --- | --- | --- | --- | --- | --- | --- | --- | --- | --- | --- | --- | --- | --- | --- | --- | --- | --- | --- | --- | --- | --- | --- |
| **P-value COMBI** |  | |  | |  |  | |  | |  |  |  | |  | 2.5x10^-10 s^ | | 8.3x10^-9 s^ | | 3.5x10^-8 s^ | 4.9x10^-12 s^ | 6.5x10^-12 s^ | | 7.7x10^-12 s^ |  |
| **P-value** | 1,4x10^-24^ | | 1.96x10^-22^ | | 2.84x10^-22^ | 5.03x10^-22^ | | 2.34x10^-12^ | | 1,6x10^-6^ | 1.91x10^-6^ | 5.614x10^-6^ | | 5.766x10^-6 r^ | 3x10^-3 s^ | | 1.2x10^-2 s^ | | 4.2x10^-4 s^ | 0.001485 ^r^  4.4x10^-4 s^ | 0.001034^r^  4.1x10^-4 s^ | | 0.001034 ^r^  4.1x10^-4 s^ |  |
| **Replication cohort** | N=287 from the Rotterdam study extended cohort  ^a^ | | | | | | | | | | | | | | | | | | | | | | |  |
| **Other significant SNPs in the region** | 30 | | | | | | | | | 15 other significant SNPs in *CYP2C9, CYP2C18, CYP2C19* and *CYP2C8* regions | | | | |  | |  | |  |  |  | |  | 10 |
| **P-value MULTI** | 2x10^-123 r^ | | 5.61x10^-106 r^ | | 7.82x10^-104 r^ | 3.08x10^-103 r^ | | 1.4x10^-45 r^ | | 3,3x10^-24 r^ | 8x10^-24 r^ | 6,44x10^-12 r^ | | 1,3x10^-11 r^  1,5x10^-4 s^ | 7x10^-5 r^  2x10^-8 s^  9.8x10^-9 t^ | | 1.9x10^-7 s^  9.3x10^-8 t^ | | 5.6x10^-6 s^  3.3x10^-6 t^ | 8.06x10^-9^ ^r^  1.9x10^-9^ ^s^  1x10^-6 t^ | 1.09x10^-8^ ^r^  2.5x10^-9 s^  1.2x10^-6 t^ | | 1.23x10^-8^ ^r^  3x10^-9 s^  1.4x10^-6 t^ |  |
| **P-value**  **UNI** |  | |  | |  |  | |  | |  |  |  | |  |  | |  | |  |  |  | |  | >10^-15 r^ |
| **Gene** | *STX4 (downstream* 60 kb *VKORC1) MYST1* *(*upstream 30kb *VKORC1*) | | *MYST1 (*upstream 30kb *VKORC1*) | | *MYST1 (*upstream 30kb *VKORC1*) | *BCKDK*  *(*upstream 20kb *VKORC1*) | | *VKORC1* | | *CYP2C9* | *CYP2C9* | *CYP2C9* | | *Downstream 6kb CYP2C19* | *CYP4F2* | | *CYP4F2* | | *Downstream 10kb CYP4F2* | *Upstream 30kb CYP2C18* | *Upstream 15kb CYP2C18* | | *Upstream 40kb CYP2C18* | *STX4 (downstream* 60 kb *VKORC1)* |
| **HGVS Names** | *NC_000016.10:g.31036758C>T* | | NC_000016.10:g.31126391C>T | | *NC_000016.10:g.31118621C>T* | NC_000016.10:g.31091000C>T | | *NC_000016.10:g.31091000C>T* | | *NC_000010.10:g.96707202C>T* | NC_000010.10:g.96725535A>C | *NC_000010.10:g.96741053A>C* | | rs3862009 | rs2108622 | | rs2074901 | | rs12610189 | rs1998591 | rs2104543 | | rs12772169 | rs10871454 |
| **SNPs** | rs10871454  (in LD with rs889548, rs1978487, rs749767, rs7294, rs9934438). | | rs889548 | | rs1978487 | rs749767 | | rs7294 | | rs4086116  (in LD with rs4917639) | rs4917639 | rs1057910 (*CYP2C9*3*) | | rs3862009 | rs2108622 | | rs2074901 | | rs12610189 | rs1998591 | rs2104543 | | rs12772169 | rs10871454 |
| **Discovery cohort** | N=1451 Caucasian subjects from the Rotterdam study | | | | | | | | | | | | | | | | | | | | | | | |
| **Treatment** | ACE | | | | | | | | | | | | | | | | | | | | | | | |
| **Ref** | [11] | | | | | | | | | | | | | | | | | | | | | | | |
| **Outcome analyzed** | | PHEN maintenance dose | | | | | | | | | Peak and trough dabigatran concentrations | | | | | | | | | Risk of major bleeding | |  |  |  |
| **P-value COMBI** | | 2.1x10^-22^ ^r^ | | 2.1x10^-22^ ^r^ | | | .9x10^-5^ ^r^ | | 2.3x10^-4^ ^r^ | |  | |  | | |  | |  | |  | |  |  |  |
| **P-value** | | 2.5x10^-3^ ^r^ | | 2.5x10^-3^ ^r^ | | | 0.39 ^r^ | | 0.71 ^r^ | |  | |  | | |  | |  | |  | |  |  |  |
| **Replication cohort** | | N=42 participants from the Rotterdam (RS-II) study ^a^ | | | | | | | | | No | | | | | | | | |  | |  |  |  |
| **Other significant SNPs in the region** | | 30 | | | | |  | |  | | 1 SNP with p-value < 5x10^-7^ | | | | | 6 SNPs with p-values < 5x10^-7^ | | 1 SNP with p-value < 5x10^-7^ | |  | |  |  |  |
| **P-value MULTI** | | 1.4x10^-18^ ^r^ | | 1.4x10^-18^ ^r^ | | | 5.3x10^-8 r^ | | 7.9x10^-8^ ^r^ | | 1.2x10^-8 v^  (TC) | | 3.2x10^-8 v^  (PC)  1.2x10^-7 v^  (TC) | | | 8.2x10^-8 v^  (PC) | | 1.7x10^-8 v^  (TC) | | 7x10-5 ^v^ | |  |  |  |
| **P-value**  **UNI** | |  | |  | | |  | |  | |  | |  | | |  | |  | |  | |  |  |  |
| **Gene** | | *STX4 (downstream* 60 kb *VKORC1)* | | *ZNF646 (downstream* 65kb *VKORC1*) | | | *Upstream 20kb CKS2* | | *Upstream 40kb SHC3* | | *CES1* | | *CES1* | | | *ABCB1* | | *CES1P2* | | *CES1* | |  |  |  |
| **HGVS Names** | | *NC_000016.10:g.31036758C>T* | | *NC_000016.10:g.31025699A>T* | | | NC_000009.11:g.91899245T>C | | *NC_000009.11:g.91837560C>T* | | *NC_000016.10:g.55810697G>T* | | *NC_000016.10:g.55827882A>G* | | | *NC_000007.13:g.87163049C>T* | | *NC_000016.10:g.55734728T>C* | | NC_000016.10:g.55810697G>T | |  |  |  |
| **SNPs** | | rs10871454 | | rs11150604  (r^2^ =1 with rs10871454) | | | rs1980889 | | rs746357 | | rs2244613 | | rs8192935 | | | rs4148738 | | rs4580160 | | rs2244613 | |  |  |  |
| **Discovery cohort** | | N=202 (Rotterdam (RS-I) study) ^a^ | | | | | | | | | N=1490 participants ^u^ | | | | | | | | | | |  |  |  |
| **Treatment** | | PHEN | | | | | | | | | DAB | | | | | | | | | | |  |  |  |
| **Ref.** | | [63] | | | | | | | | | [12] | | | | | | | | | | |  |  |  |
