# Supplementary material for: Pharmacogenetic studies with oral anticoagulants. Genome-wide association studies in vitamin K antagonist and direct oral anticoagulants
Source: Oncotarget. 2018 Jun 26;9(49):29238–58. doi: 10.18632/oncotarget.25579 (PMC6044386; doi:10.18632/oncotarget.25579)
Supplement: Supplementary file 3 [file oncotarget-09-29238-s003.docx]

| **Event** **ratios** | **Death** | 1.9 /100 patient-years | 3.60  6.07  /100 patient-years for VKA |  |  | RR (EDO 30 mg vs VKA): 0.88  RR (EDO 60 mg vs VKA): 0.89 |
| --- | --- | --- | --- | --- | --- | --- |
|  | **Intracerebral bleeding** | 0.4 /100 patients-  years |  | HR= 0.44  (DAB 150mg vs WAR) | 0.40/100 patients-years | RR (EDO 30 mg vs VKA): 0.31  RR (EDO 60 mg vs VKA): 0.46 |
|  | **GI bleeding** | 0.9 /100 patients-years |  | HR= 1.23 (DAB 150mg vs WAR) | 2.4/100 patients-years |  |
|  | **Major bleeding** | 2.1 /100 patients-years | 3.93  5.61  /100 patients-years |  | 3.32/100 patients-years |  |
|  | **Systemic embolism** | 0.1 /100 patient-years |  |  |  | RR (EDO 30 mg vs VKA): 1.26  RR (EDO 60 mg vs VKA): 0.66 |
|  | **Ischemic stroke** | 0,5% of patients | 1.65  2.85  /100 patients-years | HR= 0.92 (DAB 150mg vs WAR) |  | RR (EDO 30 mg vs VKA): 1.42  RR (EDO 60 mg vs VKA): 1.00 |
|  | **Hemorrhagic stroke** | 0,5% of patients |  |  |  |  |
|  | **Stroke** | 0.7 /100 patients-years |  |  |  | RR (EDO 30 mg vs VKA): 1.14  RR (EDO 60 mg vs VKA): 0.89 |
|  | **Stroke, TIA and systemic embolism** | 0.8 /100 patients-years |  |  |  |  |
|  | **Nº patients** | 6,785 | 210,279  501,019 | 348,750 (56.6% WAR NU, 40.2% DAB 150 mg NU and 3.2% DAB 110 mg NU). | 51,533 | 203,394 |
|  | **Indication** | NVAF | AF | NVAF | NVAF | NVAF |
|  | **Drugs** | RIV | DAB  VKA | DAB  WAR | RIV | EDO 30mg or 60mg vs dose adjusted VKA |
|  | **Reference** | [118][96][82] | [82] | [77] | [93] | [104] |
|  |  | **Prospective studies** | **Meta-analyses** | | | |

| **Event ratios (Cont.)** | **Death** | 32.6  37.8  /1000 patients-years | 3.13  5.24  /100 patients-years |  |
| --- | --- | --- | --- | --- |
|  | **Intracerebral bleeding** | 2.4  7.3  /1000 patients-years | 0.27  0.56  /100 patients-years | 0.46/100 patients-years  DAB 110 mg (VKA naïve)  0.69/100 patients-years  DAB 110 mg (VKA exp)  0.23/100 patients-years  DAB 150 mg (VKA naïve)  0.29/100 patients-years  DAB 150 mg  (VKA exp)  0.98/100 patients-years  WAR (VKA naïve)  0.71/100 patients-years  WAR (VKA exp) |
|  | **GI bleeding** | 34.2  26.5  /1000 patients-years | 2.54  2.37  /100 patients-years | 0.42/100 patients-years  DAB 110 mg (VKA naïve)  0.97/100 patients-years  DAB 110 mg (VKA exp)  0.49/100 patients-years  DAB 150 mg (VKA naïve)  0.43/100 patients-years  Dab 150 mg (VKA exp)  0.58/100 patients-years  WAR (VKA naïve)  0.51/100 patients-years  WAR (VKA exp) |
|  | **Major bleeding** | 42.7  43.9  /1000 patients-years | 3.08  3.70  /100 patients-years | 3.7/100 patients-years  DAB 110 mg (VKA naïve)^a^  3.5/100 patients-years  DAB 110 mg (VKA exp)^b^;  2.2/100 patients-years  DAB 150 mg (VKA naïve);  2.1/100 patients-years  DAB 150 mg (VKA exp);  3.7/100 patients-years  WAR (VKA naïve);  2.6/100 patients-years  WAR (VKA exp) |
|  | **Systemic embolism** |  |  |  |
|  | **Ischemic stroke** | 11.3  13.9  /1000 patients-years |  |  |
|  | **Hemorrhagic stroke** |  | 0.85  1.07  /100 patients-years |  |
|  | **Stroke** |  | 0.08  0.25  /100 patients-years |  |
|  | **Stroke, TIA and systemic embolism** |  | 0.92  1.32  /100 patients-years |  |
|  | **Nº patients** | 67,494 (≥75 years)  273,920 (≥75 years) | 12,793  12,793 | 33,945  (11,315 DAB vs 22,630 WAR) |
|  | **Indication** | NVAF | NVAF | AF |
|  | **Drugs** | DAB  WAR | DAB  WAR | DAB 110 mg  DAB 150 mg  VKA |
|  | **Reference** | Medicare FDA database [83] | The US Department of Defense Military Health System database  [84] | The Danish registry  [85] |
|  |  | **Datasets** | | |

| **Event ratios (Cont.)** | **Death** |  |  |  |  | 5.1% for day 30  and 6.3% for day 90 | 3.5/100 patient-  years |
| --- | --- | --- | --- | --- | --- | --- | --- |
|  | **Intracerebral bleeding** | 7,5% of the major bleedings | 0.49 % per year  0.96 % per year | 0.1 % per year  0.29 % per year | 0.71/100 patients-years  EDO 30mg  0.21/100 patients-years  EDO 60mg  0.44/100 patients-years  WAR |  |  |
|  | **GI bleeding** | 88,5% of the major bleedings |  |  |  |  |  |
|  | **Major bleeding** | 2.86 / 100 patients-years |  |  |  | 3.1 per 100-patient years (AF) and 4.1 per 100-patient years (VTE) | 3.0 /100-patient years  4.5 RIV 15mg 2.4 RIV 20mg /100 patient-  years |
|  | **Systemic embolism** |  |  |  |  |  |  |
|  | **Ischemic stroke** |  | 0.54% per year  0.83% per year | 0.69 % per year  1.58 % per year |  |  |  |
|  | **Hemorrhagic stroke** |  |  |  |  |  |  |
|  | **Stroke** |  |  |  |  |  |  |
|  | **Stroke, TIA and systemic embolism** |  |  |  | 0.57/100 patients-years  EDO 30mg  0.42/100 patients-years  EDO 60mg  0.53/100 patients-years  WAR |  | 1.7/100  patient-years  2.7 RIV 15mg 1.25 RIV 20mg /100 patient-  years |
|  | **Nº patients** | 27,467 | 11,411  11,411 | 1,039  1,039 | 133,733 | 1,776 | 1,204 |
|  | **Indication** | NVAF | AF | NVAF | NVAF | Stroke prevention in AF and VTE | AF |
|  | **Drugs** | RIV | RIV  WAR | RIV  WAR | EDO  WAR | RIV | RIV |
|  | **Reference** | Military Health System (Post-Marketing Surveillance Study)  [99] [96] | US Truven Health Marketscan (Revisit-US)  [96] | IMS disease analyzer database  [96][100] | Danish nationwide  [105] | Dresden NOAC  [95] | Dresden NOAC  [98] |
|  |  | **Datasets** | | | | **Registries** | |
